# *Chukrasia tabularis* limonoid plays anti-inflammatory role by regulating NF-*κ*B signaling pathway in lipopolysaccharide-induced macrophages

**DOI:** 10.29219/fnr.v67.9383

**Published:** 2023-07-14

**Authors:** Jinhuang Shen, Fan Cao, Zhiyong Huang, Xinhua Ma, Nana Yang, Haitao Zhang, Yonghong Zhang, Zhiqiang Zhang

**Affiliations:** 1 Fujian Provincial Key Laboratory of Natural Medicine Pharmacology, Department of Pharmacy, Fujian Medical University, Fuzhou, China; 2 Department of Plastic Surgery, Dermatology Hospital of Fuzhou, Fuzhou, China

**Keywords:** Chukrasia tabularis, limonoids, NO, NF-κB, anti-inflammation

## Abstract

**Background:**

*Chukrasia tabularisis,* a well-known tropical tree native to southeastern China, has anti-inflammatory and antioxidant activities, and contains large amounts of limonoids and triterpenoids.

**Objective:**

The aim of this study was to investigate the potential anti-inflammatory activity of limonoids from *C. tabularis* on lipopolysaccharide (LPS)-mediated RAW264.7 cells.

**Methods and results:**

Using a bioassay-guided approach, the chemical fraction with high anti-inflammatory activity was found and its chemical constituents were investigated. Phytochemical studies on active extracts resulted in the separation of three novel phragmalin limonoids (**1**–**3**), together with two known limonoids (**4**–**5**) and 11 tirucallane triterpenes (**6**–**16**). The activity of these isolated compounds in the production of nitric oxide (NO) on LPS-reated macrophages was evaluated. Limonoid **2** indicated significant anti-inflammatory activities with IC_50_ value of 4.58 *μ*M. Limonoid **2** notably inhibited the production of NO, interleukin- 6 and tumor necrosis factor-*α* on macrophage. Signal transduction and activation of STAT and NF-*κ*B activators were effectively blocked by limonoid **2**.

**Conclusions:**

These results indicate that limonoid **2** has an anti-inflammatory effect by the inhibiting JAK2/STAT3, iNOS/eNOS, and NF-*κ*B signaling pathways and regulating inflammatory mediators.

## Popular scientific summary

- *Chukrasia tabularis* contains large amounts of limonoids, which have anti-inflammatory and antioxidant activities.
- Three novel limonoids (**1**–**3**) were isolated from the fruits of *C. tabularis*.
- Limonoid **2** exhibited significant anti-inflammatory activities on the NO production of LPS-induced macrophages. Limonoid **2** decreased the expression level of proinflammatory cytokines in LPS-treated macrophages possibly by inhibiting STAT and NF-*κ*B signaling pathways.

Inflammation is an overreaction of the body to a stimulus and uncontrolled inflammation has been proven to be related to many diseases, such as rheumatoid arthritis, cardiovascular disease, and cancer (1–3). Glucocorticoid and non-steroidal anti-inflammatory drugs are representative anti-inflammatory medicines available in the market, but they may have serious side effects (4). In order to ameliorate these conditions, many anti-inflammatory medicinal plants have attracted attention, and a variety of plant-derived medicines have been successfully developed (5–7). Research based on medicinal plants is considered an effective strategy for developing anti-inflammatory medicines.

Limonoids are a large class of natural substances in plants, which has aroused great interest due to its complex structure and multiple biological activities (8, 9). The plants belonging to the family Meliaceae are famous for their highly diverse structure of limonoids and broad range of bioactivities (10). Chemical research on species belonging to the genus *Chukrasia* (Meliaceae) has led to separation of various phragmalin limonoids (11). Previous research has shown that phragmalin limonin has extensive bioactivity, including antifeedant (12), antibacterial (13), potassium channel blockade (14), and anti-inflammatory effects (15). As a medicinal plant, *Chukrasia tabularis* A. Juss is widely distributed in tropical Asia, including southern China (16). Its root bark has anti-influenza, astringent, and anti-diarrheal effects in Chinese medicine (17). According to reports, a variety of phragmalin limonoids with various structural characteristics have been found in the bark, seeds, and leaves of the plant (14, 18–20). In our previous studies, 14 limonoids were separated from root bark of *C. tabularis* (21), and 24 limonins were separated from the bark of *C. tabularis* (22).

The objective of this study was to investigate the potential anti-inflammatory activity of the fruits of *C. tabularis*. Phytochemical studies on active extracts resulted in the separation of three novel phragmalin limonin orthoesters (**1–3**), along with two known limonins (**4–5**) and 11 tirucallane triterpenes (**6–16**). In the study, we reported the results of separation, structure elucidation and bioassay of the separated constituents.

The anti-inflammatory assay of **1–16** on LPS-stimulated macrophages indicated that limonin **2** exhibited notable inhibitory activity, with an IC_50_ value of 4.58 *µ*M. In addition, we evaluated the effect of limonin **2** on the production of IL-6, NO and TNF-*α* by LPS-treated macrophages and its possible anti-inflammatory mechanism.

## Materials and methods

### General information

NMR spectra were acquired on a Bruker AM-400 instrument. HRESIMS was obtained in a Bruker APEX III mass spectrometer. High performance liquid chromatography (HPLC) was performed using Waters column (10 mm×250 mm). Enzyme immunoassay kit for NO, IL-6 and TNF-*α* was provided by Nanjing Jiancheng Biotech (Nanjing, China). Lipopolysaccharide (LPS) and nitric oxide (NO) were provided by Sigma (Chemical Company in St. Louis, USA). Antibodies against JAK, STAT, NF-*κ*B P65, IKB*α*, IKK*α*, iNOS, and eNOS were provided by Cell Signaling Technology (USA). Electronic circular dichroism (ECD) spectra were measured at JASCO J-1500 (J-1500, Japan) spectropolarimeter.

### Plant materials

The fruits of *Chukrasia tabularis* A. Juss were harvested in October 2018 in Fuzhou, Fujian, China. The plant was authenticated by Dr. YHZ. A voucher specimen (ZYH20181002) was deposited in the Pharmacy Department, Fujian Medical University.

### Chromatographic separation

The chipped dried fruit of *C. tabularis* (9.7 kg) was extracted with methanol. The methanol extract was partitioned by n-hexane, CH_2_Cl_2_, ethyl acetate and n-butanol, respectively. The CH_2_Cl_2_ portion was fractionated to a silica gel column to give 8 fractions (A-H). Fraction C was fractionated to an MCI gel, further applied to Sephadex LH 20 to obtain **6**, **7**, **8**, **9**, **14**, and **15**. Fraction E was fractionated on the MCI gel to obtain 7 fractions (E1–E7). Fraction E3 was fractionated to the ODS column to give three fractions (E3a1–E3a3). Fraction E3a1 was fractionated on the silica gel to give **10** and **11**. Fraction E3a2 was fractionated on the Sephadex LH 20 and then the silica column to yield **5**. Fraction E3a3 was performed on HPLC to give **1**, **2** and **3**. Fraction F was performed on MCI gel to yield 5 fractions (F1–F5). Fraction F2 were performed on Sephadex LH 20 to obtain **12** and **13**. Fraction F3 was performed on Sephadex LH 20 and applied to the HPLC to obtain **4** and **16**.

### Assessment of anti-inflammatory activity

The inhibitory activities of compounds **1**–**16** on LPS stimulated macrophages was determined by Griess reaction. The NO content was detected by the NO kit according to the protocol described earlier (23). RAW264.7 cells obtained from the China Center for Cultivated Studies (Shanghai, China) were maintained in DMEM containing 10% FBS, and under 5% CO_2_ at 37°C. Briefly, the cells were seeded in a 96-well plate (1 × 10^5^ cells/well), after 8 h of pre-incubation under 5% CO_2_ at 37°C, compounds **1**–**16** were added to the well plate, respectively, for 12 h. Then the cell of administration group and model group were induced with LPS (2 µg/mL) and the control group was treated with DMSO as contrast. The supernatant of the cell culture were collected 24 h later.

### Detection of cytokines

After 8 h of pre-incubation under 5% CO_2_ at 37°C, limonin **2** was added to the well plate, respectively, for 12 h. Then the cell of administration group and model group were induced with LPS (2 µg/mL) and the control group was treated with DMSO as contrast. The cell supernatants of samples were collected to detect the content of IL-6 and TNF-*α* by ELISA according to manufacturer protocol. And the normal range of IL-6 and TNF-*α*ELISA kits is 3.75–120 pg/mL and 20–640 pg/mL, respectively.

### Western blot analysis

Western blot analysis was performed to observe the activities of limonin **2** on NF-*κ*B P65, JAK, STAT, IKB*α*, IKK*α*, iNOS and eNOS protein levels. Macrophages were dispersed in 6 well plates for 24 h. After incubation, the cells were processed by HFPS and treated with LPS for 24 h. After treatment, cells were harvested and lysed. Western blot analysis was based on the methods described previously (24).

### Statistical analysis

These values indicate the mean ± standard error (SEM) of three independent experiments. The difference between groups is analyzed by t-test and IBM SPSS statistical software.

## Results

### Bioassay guided separation of active constituents

**Fig. 1:**
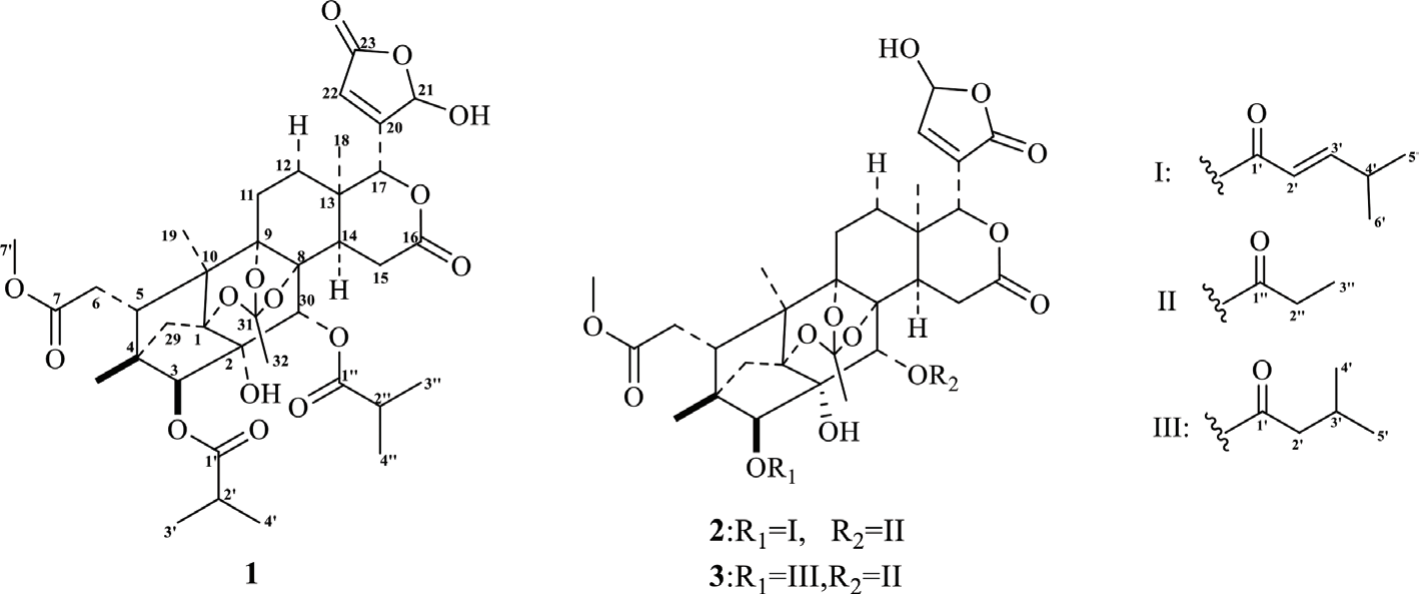
Chemical structures of compounds **1–3**.

The anti-inflammatory activity of methanol, n-hexane, CH_2_Cl_2_, ethyl acetate, and n-butanol extracts from fruits of *C. tabularis* was evaluated by xylene-induced mouse ear swelling assay. The anti-inflammatory activity assay indicated that the CH_2_Cl_2_ extract displayed stronger anti-inflammatory effect than the ethyl acetate extract (Supplementary Table S1). Therefore, separation and purification was focused on the dichloromethane extract. Five phragmalin limonoids (**1**–**5**), including three novel limonoids (**1**–**3**) and 11 tirucallane triterpenes (**6**–**16**), were isolated and identified from dichloromethane extract (Fig. 1).

### Structural elucidation

Phragmalin A (**1**) was separated as a white powder. The molecular formula C_37_H_48_O_15_ was defined based on the negative HRESI-MS and pseudo-molecular ion peak at *m/z* 731.2919 [M-H]^+^ (calcd. 731.2914), indicating 14 degrees of unsaturation. The IR spectra showed absorption, indicating the functionalities of hydroxyl (3,498 cm^-1^), ester (1,741 cm^-1^), and unsaturated carbonyl (1,696 cm^-1^). The ^1^H and ^13^C NMR spectra of 1 exhibited resonances of two 2-methylpropanoyl, three quaternary methyl, one carbomethoxy, a D ring lactone, a hemiactal group, and an ortho acetyl unit (Supplementary Table S2). The given spectral characteristics and their comparison with the simultaneous presence of spectrum 5 strongly suggested that 1 is a limonoid with a phragmalin skeleton. ^1^H and ^13^C NMR spectra for 1 were highly similar to the data for 5 in ring A, B, C, and D; the main difference in the 5 data of C-21, C-20, C-23, and C-22 in the E ring was the presence of a *γ*-hydroxybutenolactone group, rather than a furan group. In the HMBC spectra, a clear cross peak of H-18 / C-13, C-12, C-14 and C-17, H-19 /C-1, C-9, C-10 and C-5, H-28/C-3, C-5, C-4 and C-29, H-30 /C-1, C-2, C-3, C-8 and C-9 and H-15 /C-8, C-14, C-13 and C-16 were observed. Hence, the plane structure of 1 was deduced to be phragmalin 1, 8, 9, orthoacetate (25–27) (Supplementary Fig. S1), and the positions of two 2-methyl propanoyl and a methyl ester moiety were situated in C-3, C-30, and C-7, respectively. By comparing the ^13^C NMR spectra with a corresponding data model for part of Turrapubesin G (28) and 21- hydroxy- 20 (22) -ene-21, a 23-*γ*-lactone group was clearly identified. The HMBC correlation from H-22 to C-17 and from H-17 to C-20 clearly showed a connection of ring D with E. The relative configuration of C-3, C-30, and C-17 were confirmed with NOESY experiments (Supplementary Fig. S1). The ECD spectrum 1 calculated by TDDFT (29, 30) was very consistent with the experimental spectrum and pointed to the absolute configuration of 1*R*, 2*S*, 3*S*, 4*R*, 5*S*, 8*R*, 9*S*, 10*R*, 13*S*, 14*R*, 17*R*, 21*R*, and 30*R* of 1 (Supplementary Fig. S2A). Thus, the absolute configuration of 1 was characterized as depicted in Supplementary Fig. S1 and named Phragmalin A.

Chukrasin F (**2**) was obtained as a white powder. The HRESI-MS spectra of 2 indicated an ion peak at *m/z* 745.3094[M+H]^+^, corresponding to the molecular formula C_38_H_48_O_15_ (calcd. [C_38_H_49_O_15_]^+^ for 745.3070). The IR spectra indicated absorption bands of *γ*-lactone (1,743 cm^-1^) and ester carbonyl (1,720 cm^-1^) functionalities. The ^1^H and ^13^C NMR of 2 contained signals representing one propanoyl, three quaternary methyl, one carbomethoxy, one 2-ene-4-methyl-pentanyloxy, one D ring lactone, one hemiactal group, and an orthoacetyl group (Supplementary Table S2). The aforementioned spectral characteristics and the comparison with the co-occurrence of 1 strongly suggested that 2 is a limonoid with a phragmalin skeleton. The ^1^H and ^13^C NMR spectra of 2 on the B, C, and D rings were very similar to 1, and the main difference between C-21, C-20, C-23, C-22, and compound 1 on the E ring was the presence of the *γ*-hydroxy butenolide group, as well as a propanoyloxy and a 2- ene-4-methyl-pentanyloxy moiety on the B and A rings at C-30 and C-3. The *γ*-hydroxybutenolide unit was proposed according to the four carbon atoms and the corresponding proton. The 23- hydroxy-20(22)-ene-23, 21-*γ*-lactone group can be clearly observed by comparing the chromatographic values with the corresponding values model for part of the compound 17- (5-methoxy- 2-oxofuran- 3- yl)- 28- deoxonimbolide (31). In the NOESY spectra, the correlation of H-28 with H-5, H-5 with H-30, and H-28 with H-30 showed that H-28, H-30, and H-5 were all *β*-oriented. Furthermore, H-29 *pro-s* combined with H-3 showed that H-3 was *α*-oriented. Thus, the *α*-orientation of C-17 of the *γ*-hydroxy butenolide group was established. The experimental spectrum was very constant with the calculated spectra (Supplementary Fig. S2B), indicating that compound 2 has (1*R*, 2*S*, 3*S*, 4*R*, 5*S*, 8*R*, 9*S*, 10*R*, 13*S*, 14*R*, 17*R*, 23*S*, and 30*R*) an absolute configuration.Thus, the absolute configuration of 2 was constructed as shown in Supplementary Fig. S5.

Chukrasin G (**3**) was found to have the molecular formula C_37_H_48_O_15_, which was in agreement with the [M]^+^ ion peak 732.2974 (calcd. 732.2992) in HRESI-MS. The molecular formula of 3 was 12 mass units less than compound 2, which is consistent with the loss of a carbon atom. The ^1^H and ^13^C NMR spectrum of 3 exhibited high similarity to 2 (Supplementary Table S2). The only difference was the presence of a 3-methylbutyryl moiety at C-3 of 3, substituted for a 2-ene-4-methyl-pentanyloxy moiety in 2 (Fig. 1). According to the similar ^1^H and ^13^C NMR chemical shift, the relative configuration of 3 was the same as that of the co-occurrence of 2. The calculated ECD spectra 3 were in agreement with the experimental curves (Supplementary Fig. S2C). Thus, the absolute configuration was designated as 1*R*, 2*S*, 3*S*, 4*R*, 5*S*, 8*R*, 9*S*, 10*R*, 13*S*, 14*R*, 17*R*, 23*S,* and 30*R* of 3 (Supplementary Fig. S8).

The known compounds, phragmalin, 22, 23-dihydro-23-hydroxy-21-oxo-3, 30- di- isobutyrates (**4**) (25), phragmalin, 3, 30-di-isobutyrates (**5**) (25), bourjotinolone A (**6**) (32), 21*β*-methoxy-25-ene-bitrinonediol (**7**) (33), (13a, 14b, 17a, 20S, 23R, 24R)- 23, 24-dihydro-xylanosta-7, 25-dien-3-one (**8**) (34), 21*β*- methylmelianodiol (**9**) (35), 24-hydroxyl-7, 25-dien-3-one (**10**) (36), melianodiol (**11**) (35), 21*α*, 25- dimethyl- melianodiol (**12**) (37), 21*β*, 25- dimethylmelianodiol (**13**) (37), 3R, 23S, 24R, 25- tetraol- 7- glyoxiene (**14**) (38), piscidinol A (**15**) (39), andhispidone (**16**) (32), were determined by interpretation of their spectral data and comparison with literature.

### Cytotoxic activity of compounds 1–16

The cytotoxicity of the separated compounds was assessed against seven tumor cell lines, Huh7, HepG2, KB, H460, Hela, A-549 and MCF-7 (40, 41). The phragmalin limonoids **1**-**5** indicated weak cytotoxicity on these tumor cell lines, while tirucallane triterpenoids **6**, **9**, and **11** showed potent inhibitory activities on seven human tumor cell lines. Tirucallane triterpenoid **7** showed moderate cytotoxicity to these seven cancer cell lines. Tirucallane triterpenoids (**6**–**16**) showed stronger cytotoxicity than the phragmalin limonoids (**1**–**5**) (Supplementary Table S3).

### Effect of 1–16 on NO production

In order to find anti-inflammatory components, we tested the effect of all the isolates on the NO production of LPS-induced macrophages. The results of **1**–**16** producing NO inhibitory activity on RAW264.7 cells are shown in Table 1. Compounds **1**–**16** exhibited significant to potent inhibitory activities. Phragmalin-type limonoid orthoesters (**1**–**5**) exhibited potent anti-inflammatory effects, especially limonoids **1**–**3**, with inhibition rates between 4.58 and 10.45 *μ*M, indicating that the orthoester groups in phragmalin limonoids play a crucial role in anti-inflammation. Tirucallane triterpenoids (**6**, **8**–**15**) showed potent to moderate anti-inflammatory effect, with inhibition rate ranging from 11.15 to 95.09 *μ*M, indicating that phragmalin limonoids have stronger anti-inflammatory activity than tirucallane triterpenoids. Limonin **2** showed the strongest anti-inflammatory effect, with an IC_50_ value of 4.58 *μ*M. Therefore, we further investigated the potential anti-inflammatory effect and molecular mechanisms of limonin **2**.

**Table 1:** Inhibitory activity of compounds 1–16 on LPS-induced NO production in RAW264.7 cells

Compounds	IC_50_(*μ*M)	Compounds	IC_50_(*μ*M)
**1**	10.29 ± 0.39	**10**	95.09 ± 9.40
**2**	4.58 ± 0.72	**11**	13.34 ± 2.67
**3**	10.45 ± 0.98	**12**	13.93 ± 5.33
**4**	21.92 ± 5.61	**13**	24.86 ± 4.60
**5**	35.60 ± 7.23	**14**	11.15 ± 1.12
**6**	35.60 ± 4.29	**15**	33.56 ± 4.77
**7**	>100	**16**	>100
**8**	46.07 ± 6.11	**Indomethacin^a^**	26.18 ± 1.56
**9**	52.52 ± 8.98		

a Positive control

### Effect of limonin 2 on RAW264.7 cell viability

**Fig. 2:**
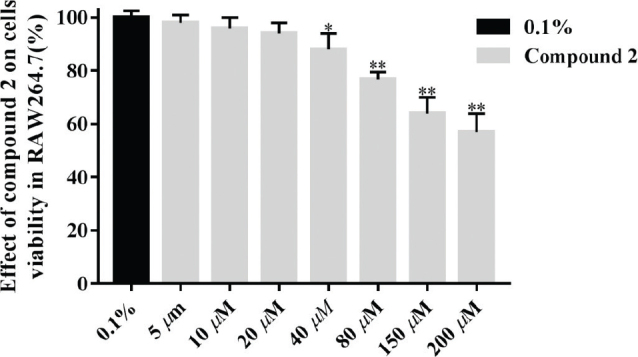
Effect of compound **2** on RAW264.7 cells viability. RAW264.7 cells were treated by assigned concentration of compound **2** or DMSO for 24 h. The data are expressed as the mean ± SEM (*n* = 3). The cells viability (%) = (OD_compound_ **_2_** – OD_DMSO_)/OD_DMSO_ ×100. *P < 0.05,**P < 0.01 compared with control cells treated by DMSO. Statistical differences were analyzed by unpaired *t*-test.

The cytotoxicity of limonin **2** to RAW264.7 cells was detected by SRB assay. After treatment with limonin **2** with 5 ~ 200 *μ*M for 24 h, the relative survival rate was 100.7, 96.3, 93.9, 88.0, 76.4, 64.4, and 56.7%, respectively (Fig. 2), which indicates that the viability of RAW264.7 cells was not notably affected by compound **2** within 24 h and the concentration was 0–20 *μ*m. Therefore, compound **2** of 0–20 *μ*m was used in subsequent experiments to avoid the influence of cytotoxic and anti-inflammatory effect detection.

### Effect of 2 on LPS treated production of IL-6 and TNF-α

**Fig. 3:**
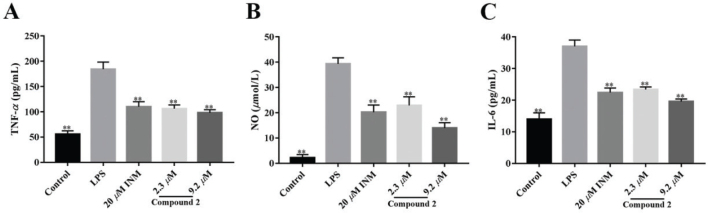
Effects of compound **2** on the production of TNF-*α*, NO and IL-6 in LPS-induced macrophages. The macrophages were incubated with LPS (1 *μ*g/mL) and treated with compound **2** (2.3 and 9.2 *μ*mol/L) for 24 h. Indomethacin (INM) was used as the positive control. (A) The levels of TNF-*α*, (B) NO, and (C) IL-6 in the supernatant were assayed using ELISA kits. All values given are the mean ± SEM. *P <0.05, **P <0.01 compared with the LPS group.

The anti-inflammatory activity of limonin **2** on LPS treated IL-6, NO and TNF-*α* was detected (42). As shown in Fig. 3A, B and C, the addition of limonin **2** notably inhibited the production of NO, IL-6 and TNF-*α*. The result indicated that limonin **2** can inhibit the expressions of IL-6, TNF-*α* and NO in LPS-treated macrophages, and achieve an anti-inflammatory effect. The inhibitory activity of limonin **2** is better than that of indomethacin, indicating that limonin **2** could effectively inhibit the inflammatory response of LPS-treated macrophages.

### Compound 2 blocked LPS-treated NF-κB activation

**Fig. 4:**
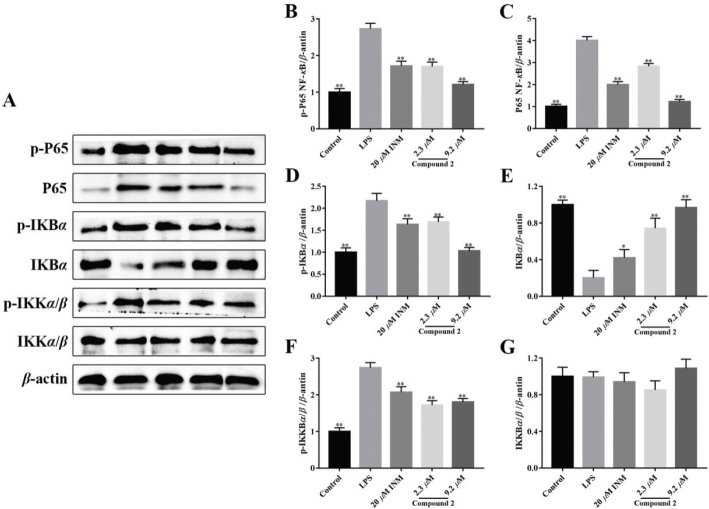
Effect of compound **2** on inhibiting activation of the IKK*β*/NF-*κ*B signaling pathway induced by LPS. (A) Representative result of Western blot. Relative expression levels of p-P65(B), P65(C), p-IKB*α* (D), IKB*α* (E), p-IKK*α*/*β*(F), and IKK*α*/*β*(G). *P < 0.05, **P < 0.01 compared with model cells that treated by LPS. Statistical differences were analyzed by unpaired *t*-test.

The NF-*κ*B signaling cascade plays an important role in the adjustment of inflammation (43, 44). The expression of NF-*κ*B P65, IKB, and IKK*α* were measured by Western blot to investigate the anti-inflammatory mechanism of limonin **2**. The results exhibited that limonin **2** downregulates the expressions of NF-*κ*B p-P65 (Fig. 4A, B and C). Limonin **2** inhibited the phosphorylation and degradation of IKB*α* (Fig. 4A, D and E) and IKK*α* (Fig. 4A, F and G). These data indicate that limonin **2** blocks LPS-stimulated activation of the NF-*κ*B signaling.

### Compound 2 blocked LPS-treated STAT3 activation

**Fig. 5:**
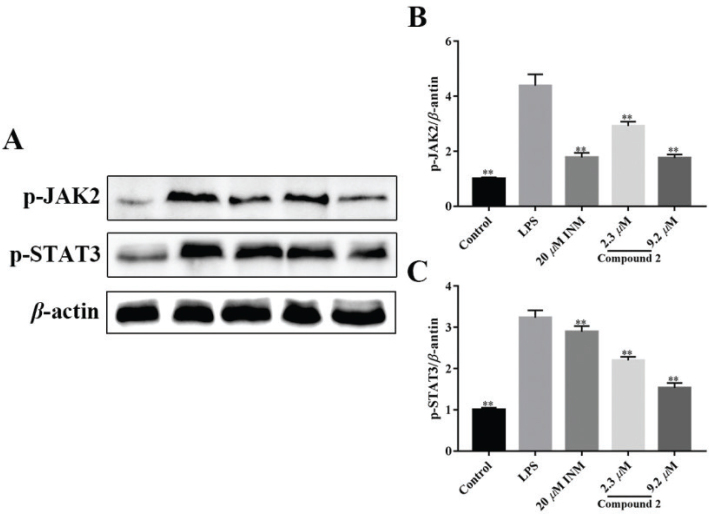
Effect of compound **2** on inhibiting activation of the JAK2/STAT3 signaling pathway induced by LPS. (A) Representative result of Western blot. Relative expression levels of p-JAK2 (B) and p-STAT3(C). *P < 0.05, **P < 0.01 compared with model cells that are treated by LPS. Statistical differences were analyzed by unpaired *t*-test.

To study the effect of limonin **2** on the STAT signaling cascades of LPS-treated macrophage, the levels of JAK2 and STAT3 was measured (45). As shown in Fig. 5A, B and C, the expression levels of STAT3 and JAK2 were increased notably in RAW264.7 cells compared with control group, while the expression level of STAT3 and JAK2 was notably reduced in limonin **2** group.

### Compound 2 blocked LPS-treated eNOS activation

**Fig. 6:**
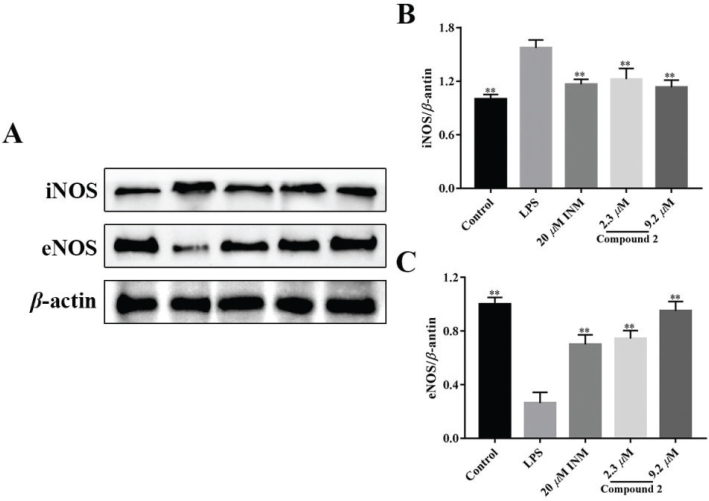
Effect of compound **2** on inhibiting activation of the iNOS/eNOS signaling pathway induced by LPS. (A) Representative result of Western blot. Relative expression levels of iNOS (B) and eNOS (C). *P < 0.05, **P < 0.01 compared with model cells that treated by LPS. Statistical differences were analyzed by unpaired *t*-test.

To study the effect of limonin **2** on eNOS signaling cascade on LPS treated macrophages (46), the level of eNOS was measured. The upregulation of iNOS expression in LPS-treated macrophage was observed (Fig. 6A and B). Compound **2** inhibited the expression of iNOS stimulated by LPS in macrophage. At the same time, downregulation of eNOS protein expression was also found in LPS-stimulated macrophages (Fig. 6A and C). Similarly, eNOS protein expression was completely restored with compound **2**. The effects of limonin **2** on expression of iNOS and eNOS in macrophages induced by LPS did not differ between doses.

## Discussion

In this study, the biological activity of different chemical fractions of a methanol extract of *C. tabularis* was evaluated. The results showed that the dichloromethane fraction showed obvious anti-inflammatory activity and its phytochemical characteristics were further analyzed. Five phragmalin limonoids (**1**–**5**), including three novel limonoids (**1**–**3**) and 11 tirucallane triterpenes (**6**–**16**), were separated and identified from dichloromethane extract. All isolated compounds (**1**–**16**) were measured for NO productions in LPS-treated macrophages and cytotoxic activity.

We used further experiments to explore the inflammatory effect of compound **2** in macrophages in vitro. The results show that compound **2** significantly reduced the secretion levels of NO, IL-6, and TNF-*α* after treating macrophages with LPS (Fig. 3A, B and C). At the same time, as the dose of compound **2** increased, the secretion level of NO, IL-6, and TNF-*α* against RAW264.7 cells gradually reduced, indicating that compound **2** helped to reduce inflammatory responses to LPS-induced macrophages.

To research the anti-inflammatory mechanism of limonin **2**, we tested NF-*κ*B signaling proteins in macrophages. The NF-*κ*B signaling cascade plays a key role in the inflammatory process (47). Research has indicated that in LPS-treated macrophages, the NF-*κ*B signaling cascades are activated (48, 49). The results indicated that the level of NF-*κ*B p-P65, IKK*α,* and IKB*α* in macrophages treated with LPS were notably increased compared with control group (Fig. 4A, B, C, D and F), which indicates that the NF-*κ*B signaling cascades in LPS-treated macrophages is notably activated. Limonin **2** treatment significantly decreased the levels of these proteins with notable anti-inflammatory activities (Fig. 4A–G). The result indicated that limonin **2** can inhibit LPS-stimulated macrophage inflammation by inhibiting the NF-*κ*B signaling cascades.

The JAK and STAT signaling cascades are two major cascades that include transcription factors associated with pro-inflammatory cytokine responses in LPS-treated macrophage inflammation (50). Proinflammatory cytokines are fundamental regulators of inflammation development (51). The improvement of cytokine and JAK and STAT expression was closely associated with the severity of LPS-treated macrophage inflammation. An important finding of our study was that compound **2** treatment reduced JAK2 and STAT3 phosphorylation, suggesting that inhibition of the JAK2/STAT3 cascade may be involved in compound **2**′s anti-LPS-induced inflammation effect, which is related to the expression of proinflammatory cytokine genes. At the molecular level, lower STAT3 signaling cascades downstream of added to the mild proinflammatory cytokines induced by compound **2**. Compound **2**′s downregulation of STAT3 may be associated with the destruction of upstream kinase JAK2.

iNOS are a sign of inflammation, previous studies showed that macrophages in the treatment of the LPS, iNOS are upregulated in the cells (52). Increased iNOS in tissue correlates with disease severity (53). Recent results have indicated that eNOS may be involved in protecting cells from inflammation. In the present study, compound **2** was found to upregulate eNOS expression and downregulate iNOS expression in macrophages, indicating that compound **2** could inhibit apoptosis and exert biological effects, which is positively correlated with the concentration.

Inflammatory bowel disease is caused by excessive immune system activation and multiple signaling pathways are involved in the innate and adaptive immune response processes. The JAK/STAT signaling pathway and the NF-*κ*B pathway, for example, are cytokine transport hubs and their activation is critical for the regulation of inflammatory response (54). At the moment, most anti-inflammatory drugs for IBD work by regulating the immune system, with Chinese herbal medicines showing superior therapeutic effects and safety when compared with synthetic drugs. Many traditional Chinese medicines have been shown in studies to relieve disease by regulating the JAK/STAT signaling pathway and the NF-*κ*B pathway (55, 56). We discovered that limonin **2** has anti-inflammatory properties and plays anti-inflammatory role by regulating NF-*κ*B signaling pathway in LPS-induced macrophages. Further studies are needed to determine the therapeutic efficacy, effective therapeutic dose and potential toxicity of limonin **2** in animal models of UC. Further research is expected to provide a better understanding of limonin **2**’s ability to treat UC.

## Conclusions

A total of 16 compounds were isolated by screening the anti-inflammatory activity of extracts from *C. tabularis* fruits, including three novel limonins, two known limonins and 11 triterpenoids. The data provided indicate that compound **2** can effectively inhibit the inflammatory responses by inhibiting the activation of STAT3, iNOS/eNOS and NF-*κ*B signaling pathways. Therefore, compound **2** may be a valid agent for the development of anti-inflammatory medicines and can be determined as the source of natural anti-inflammatory molecule.

## Conflict of interest and funding

The authors declared that there is no conflict of interest. This work was financed by the National Natural Science Foundation of China (41806173), Fujian Natural Science Foundation (2020J01619 and 2018J01847), Fujian Key Laboratory of Natural Medicine Pharmacology of Fujian Medical University (FJNMP-202202).

## Authors’ contributions

Jinhuang Shen, Fan Cao, Zhiqiang Zhang and Yonghong Zhang conceived and designed the experiments and prepared the manuscript; Zhiyong Huang, Xinhua Ma, Nana Yang, and Haitao Zhang conducted experiments and analyzed the data.
