# Supplementary material for: Chukrasia tabularis limonoid plays anti-inflammatory role by regulating NF-κB signaling pathway in lipopolysaccharide-induced macrophages
Source: Food Nutr Res. 2023 Jul 14;67:10.29219/fnr.v67.9383. doi: 10.29219/fnr.v67.9383 (PMC10392864; doi:10.29219/fnr.v67.9383)
Supplement: Supplementary file 1 [file FNR-67-9383-s001.doc]

**List of Supporting Information**

Ear edema induced by xylene in mice

Table S1 Effects of *Chukrasia tabularis* on ear edema induced by xylene in mice.

Cytotoxicity bioassays

NMR and ESI-MS spectroscopic data

Table S2 ^1^H-NMR(400 MHz) and ^13^C-NMR(100 MHz) spectral data of compounds**1**-**3**

Table S3 Cytotoxicity of compounds 2-16 against tested cell lines

Figure S1 Key HMBC and NOESY of compound **1**

Figure S2 Calculated and experimental ECD spectra of **1-3**.

Figure S3 ^1^H NMR spectrum (400 MHz) of compound **1** in CDCl_3_.

Figure S4 ^13^C NMR spectrum (100 MHz) of compound **1** in CDCl_3_.

Figure S5 Key HMBC and NOESY of compound **2.**

Figure S6 ^1^H NMR spectrum (400 MHz) of compound **2** in CD_3_OD.

Figure S7 ^13^C NMR spectrum (100 MHz) of compound **2** in CD_3_OD.

Figure S8 Key HMBC and NOESY of compound **3.**

Figure S9 ^1^H NMR spectrum (400 MHz) of compound **3** in CD_3_OD.

Figure S10 ^13^C NMR spectrum (100 MHz) of compound **3** in CD_3_OD.

**Ear edema induced by xylene in mice**

Male ICR mice (18–20 g) were provided by the Laboratory Animal Center, Fujian Medical University. After intragastric administration of the extract or control for 1 hour, each mouse’s right ear was treated with 40 *μ*L xylene solution, and the left ear was taken as the control. One hour after xylene treatment, the mice were sacrificed by cervical dislocation. The round part of each ear, with a diameter of 6mm, was weighed by an electronic analytical balance with an accuracy of 0.1 mg, and the inhibitory effect on ear edema was calculated. The weight difference of two plugs was used to measure the edema response (Table S1). Animal care and use complied with institutional guidelines, and the program was affirmed by the Experimental Animal Management Committee of Fujian Medical University.

**Table S1** Effects of *Chukrasia tabularis* on ear edema induced by xylene in mice.

| Extracts/Fractions | Dose (mg/kg) | Edema Degree ($\overline{\text{x}}$±SD, mg) | Inhibition Rate (%) |
| --- | --- | --- | --- |
| Control group |  | 7.83±0.36 |  |
| Aspirin | 200 | 4.99±0.30^*^ | 36.30 |
| Methanol extract | 400 | 5.30±0.36^*^ | 32.27 |
| Petroleum ether fraction | 400 | 5.12±0.59^*^ | 34.62 |
| Dichloromethane fraction | 400 | 4.48±0.25^*^ | 42.78 |
| Ethyl acetate fraction | 400 | 4.70±0.34^*^ | 40.03 |
| n-Butanol fraction | 400 | 5.26±0.24^*^ | 32.84 |

^*^*P* < 0.01 *vs.* Control group

**Table S2 ^1^H-NMR(400 MHz) and ^13^C-NMR(100 MHz) spectral data of compounds1-3**

| No. | **1**^a^ | | **2**^b^ | | **3**^b^ | |
| --- | --- | --- | --- | --- | --- | --- |
|  | *δ*_H_ (J inHz） | *δ*_C_ | *δ*_H_ (J inHz） | *δ*_C_ | *δ*_H_ (J inHz） | *δ*_C_ |
| 1 |  | 86.9 |  | 86.9 |  | 87.5 |
| 2 |  | 79.7 |  | 79.5 |  | 81.0 |
| 3 | 4.19(s) | 82.7 | 4.63 (s) | 83.2 | 4.51 (s) | 84.7 |
| 4 |  | 45.4 |  | 45.5 |  | 46.1 |
| 5 | 2.46 (dd, 12.8, 5.8) | 37.1 | 2.90 (dd, 14.4, 7.3) | 37.2 | 2.21 (m) | 39.6 |
| 6 | 2.10(m) | 34.0 | 2.62 (dd, 15.9, 7.6) | 34.4 | 2.84(dd, 14.0, 7.1) | 36.9 |
|  | 2.16(m) |  | 2.91 (dd, 15.9, 7.6) |  | 2.91 (m) |  |
| 7 |  | 169.4 |  | 172.8 |  | 173.9 |
| 8 |  | 86.0 |  | 86.0 |  | 86.4 |
| 9 |  | 85.5 |  | 85.5 |  | 86.1 |
| 10 |  | 45.3 |  | 45.5 |  | 45.6 |
| 11*α* | 1.34(m) | 25.3 | 1.75 (m) | 25.4 | 1.72 (m) | 25.4 |
| 11*β* | 1.82(m) |  | 2.16 (m) |  | 2.14 (m) |  |
| 12*α* | 1.34(m) | 29.8 | 2.31 (m) | 27.9 | 1.29 (m) | 29.8 |
| 12*β* | 1.38(m) |  | 2.52 (m) |  | 1.31 (m) |  |
| 13 |  | 33.8 |  | 33.9 |  | 33.7 |
| 14 | 1.73(m) | 42.5 | 2.15 (m) | 45.4 | 2.19 (m) | 45.6 |
| 15*α* | 2.30(dd, 17.2, 10.6) | 26.4 | 2.80(dd, 16.9, 4.8) | 26.6 | 2.39 (dd, 17.2, 5.2) | 27.3 |
| 15*β* | 2.79(dd, 17.2, 10.6) |  | 3.15(dd, 16.9, 4.8) |  | 3.12 (m) |  |
| 16 |  | 169.4 |  | 169.4 |  | 170.5 |
| 17 | 5.11(s) | 78.4 | 5.60(s) | 78.5 | 5.38 (s) | 80.3 |
| 18 | 0.72(s) | 19.9 | 1.14(s) | 19.9 | 1.13 (s) | 17.8 |
| 19 | 0.77(s) | 16.4 | 1.16 (s) | 16.5 | 1.19 (s) | 15.9 |
| 20 |  | 164.0 |  | 134.3 |  | 130.1 |
| 21 | 5.79(s) | 97.7 |  | 163.9 |  | 171.1 |
| 22 | 5.83(s) | 122.0 | 7.29 (s) | 149.9 | 6.30 (s) | 122.4 |
| 23 |  | 169.1 | 6.20 (d, 5.4) | 97.7 | 6.00 (d, 5.6) | 104.1 |
| 28 | 0.51(s) | 14.5 | 0.90 (s) | 14.6 | 0.89 (s) | 13.9 |
| 29a | 1.30(d, 10.8) | 39.6 | 1.63 (d, 10.8) | 39.7 | 1.65 (d, 10.6) | 36.9 |
| 29b | 1.55(d, 10.8) |  | 1.96 (d, 10.8) |  | 1.95 (d, 10.6) |  |
| 30 | 5.42(s) | 70.4 | 5.87(s) | 70.7 | 5.79 (s) | 71.5 |
| 31 |  | 119.2 |  | 119.2 |  | 119.6 |
| 32 | 1.25(s) | 21.2 | 1.68 (s) | 21.2 | 1.62 (s) | 20.4 |
| 1´ |  | 177.1 |  | 177.4 |  | 178.4 |
| 2´ | 2.50(m) | 34.7 | 6.29 (d, 5.4) | 122.2 | 2.27 (m) | 27.9 |
| 3´ | 0.65 (d, 6.5) | 18.1 | 7.27 (d, 4.9) | 163.8 | 2.49 (m) | 32.3 |
| 4´ | 0.67 (d, 6.5) | 19.5 | 2.16 (m) | 42.7 | 0.93 (d, 6.9) | 14.0 |
| 5´ |  |  | 1.14 (d, 7.1) | 18.1 | 0.93 (d, 6.9) | 14.0 |
| 6´ |  |  | 1.19 (d, 7.1) | 20.4 |  |  |
| 1´´ |  | 175.1 |  | 175.1 |  | 175.9 |
| 2´´ | 2.13(m) | 34.7 | 1.67 (q, 7.3) | 28.9 | 2.30 (q, 7.3) | 27.9 |
| 3´´ | 0.73 (d, 6.5) | 20.2 | 1.08 (t, 7.3) | 8.8 | 1.04 (t, 7.3) | 8.2 |
| 4´´ | 0.76 (d, 6.5) | 20.4 |  |  |  |  |
| OMe-7´ | 3.33(s) | 53.0 | 3.75(s) | 53.0 | 3.67 (s) | 56.8 |
| ^a^ Recorded in CDCl_3_, ^b^ Recorded in CD_3_OD | | | | | | |

**Cytotoxicity bioassays**

Huh-7 (hepatoma), MCF-7 (human breast), KB (oral epithelial), Hela (cervical), H460 (lung), A-549 (lung), and HepG2 (hepatocellular) cancer cell lines (provided by Shanghai Cell Bank) were maintained in RPMI 1640 at 37 ℃, supplemented with 10% FBS. Cytotoxicity was determined by the SRB method, with 5-fluorouracil (5-FU) as a positive control. The IC_50_ value was calculated using the GraphPad prism program.

**Table S3** Cytotoxicity of compounds **2-16** against tested cell lines (*μ*M) ^a^

| **Compounds** | **IC_50_ (*μ*M)** | | | | | | |
| --- | --- | --- | --- | --- | --- | --- | --- |
|  | **Huh-7** | **HepG2** | **KB** | **H460** | **Hela** | **A549** | **MCF-7** |
| **2** | 72.09±13.45 | 69.37±10.56 | 39.23±2.17 | 60.78±6.96 | 33.17±8.41 | 54.67±10.08 | 23.38±5.81 |
| **3** | >100 | 99.37±10.56 | >100 | >100 | >100 | >100 | >100 |
| **5** | >100 | 78.03±12.66 | >100 | >100 | >100 | >100 | >100 |
| **6** | 29.58±7.69 | 59.32±4.67 | 35.13±5.07 | 35.13±5.07 | 29.32±7.11 | 43.62±10.55 | >100 |
| **7** | 94.67±12.53 | >100 | 40.88±6.56 | >100 | 58.36±9.09 | >100 | >100 |
| **9** | 59.54±13.11 | 44.32±6.00 | 56.58±8.30 | 59.54±13.11 | 59.98±4.19 | 65.99±10.08 | >100 |
| **11** | 79.76±9.24 | 79.98±5.26 | 63.52±8.25 | 73.16±10.46 | 86.33±9.28 | 45.05±6.33 | 52.60±7.20 |
| **5-Fluorouracil^b^** | 36.16±4.17 | 30.49±1.54 | 19.50±1.24 | 13.69±2.85 | 2.31±0.98 | 36.16±1.73 | 23.44±1.22 |

**^a^** Compounds **4, 8, 10** and **12-16** were inactive against all cell lines tested (IC_50_>100*μ*M). **^b^** Positive control

**NMR and ESI-MS spectroscopic data**

Phragmalin A (**1**): White powder; [*α*]^28^_D_: -1.00 (*c* 2.39, CHCl_3_); UV (MeOH)210 nm; IR (KBr): *ν*_max_ 3498, 2969, 1741, 1696, 1260, 1068 cm^-1^; The ^1^H and ^13^CNMR data, listed in Table S2; HRESI-MS *m/z* 731.2919 [M-H]^+^ (calcd. for C_37_H_47_O_15_,731.2914).

Chukrasin F (**2**): [*α*]^28^_D_: -0.26 (*c* 0.40, CHCl_3_); UV (MeOH) 210 nm; IR (KBr): *ν*_max_ 3565, 2973, 1743, 1720, 1271, 1047 cm^-1^; The ^1^H and ^13^CNMR data, listed in Table S2; HRESI-MS *m/z* 745.3094 [M+H]^+^ (calcd. for C_38_H_49_O_15_, 745.3070).

Chukrasin G (**3**): [*α*]^28^_D_: -0.13 (*c* 0.24, CHCl_3_); UV (MeOH) 210 nm; IR (KBr): *ν*_max_ 3560, 2971, 1746, 1696, 1211, 1056 cm^-1^; The ^1^H and ^13^CNMR data, listed in Table S2; HRESI-MS *m/z* 756.2918 [M+Na+H]^+^ (calcd. for C_37_H_49_O_15_, 756.2968).

Figure S1 Key HMBC and NOESY of compound **1**

**
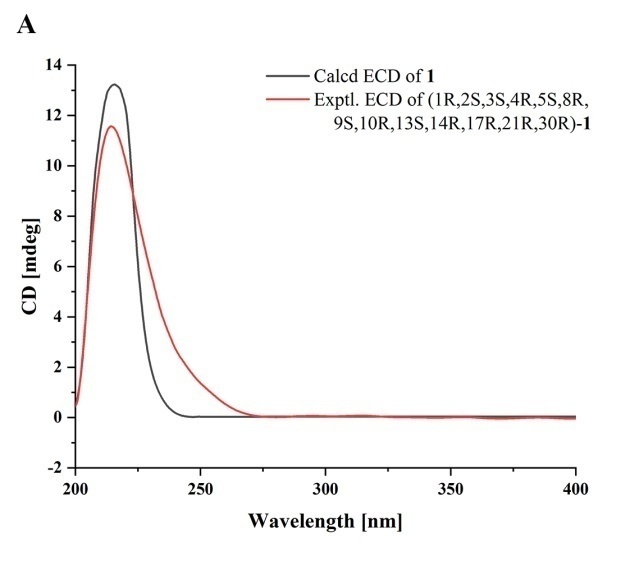
**
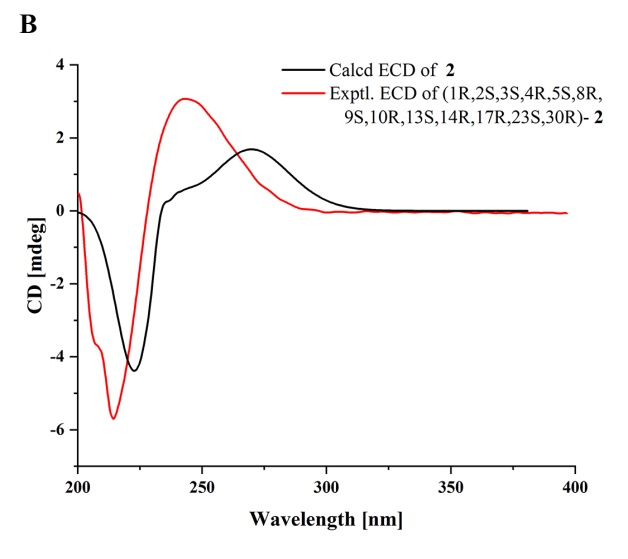

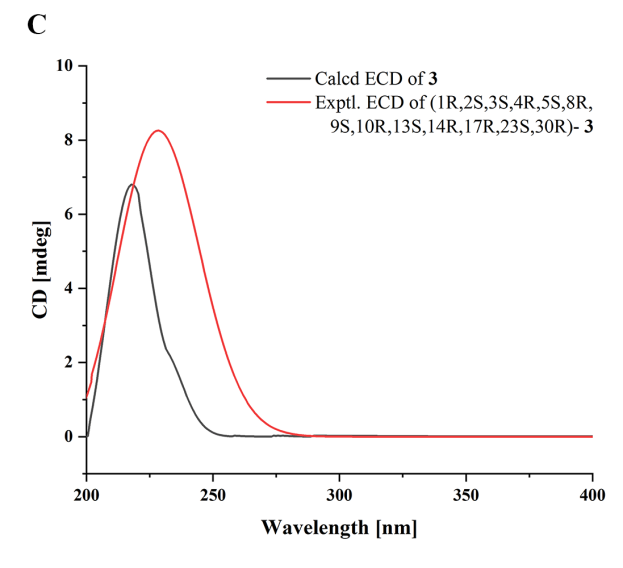


Figure S2 Calculated and experimental ECD spectra of **1-3**.

Figure S3 ^1^H NMR spectrum (400 MHz) of compound **1** in CDCl_3_.

Figure S4 ^13^C NMR spectrum (100 MHz) of compound **1** in CDCl_3_.

Figure S5 Key HMBC and NOESY of compound **2**.

Figure S6 ^1^H NMR spectrum (400 MHz) of compound **2** in CD_3_OD.

Figure S7 ^13^C NMR spectrum (100 MHz) of compound **2** in CD_3_OD.

Figure S8 Key HMBC and NOESY of compound **3.**

Figure S9 ^1^H NMR spectrum (400 MHz) of compound **3** in CD_3_OD.

Figure S10 ^13^C NMR spectrum (100 MHz) of compound **3** in CD_3_OD.
